# Proteomic Profiling Reveals Specific Molecular Hallmarks of the Pig Claustrum

**DOI:** 10.1007/s12035-023-03347-2

**Published:** 2023-04-24

**Authors:** Andrea Pirone, Federica Ciregia, Giulia Lazzarini, Vincenzo Miragliotta, Maurizio Ronci, Mariachiara Zuccarini, Lorenzo Zallocco, Daniela Beghelli, Maria Rosa Mazzoni, Antonio Lucacchini, Laura Giusti

**Affiliations:** 1 Department of Veterinary Sciences, University of Pisa, Pisa, Italy; 2 Department of Clinical and Experimental Medicine, University of Pisa, Pisa, Italy; 3 Department of Medical, Oral and Biotechnological Sciences, University G. D’Annunzio of Chieti-Pescara, Chieti, Italy; 4 Interuniversitary Consortium for Engineering and Medicine, COIIM, Campobasso, Italy; 5 Department of Translational Research and New Technologies in Medicine and Surgery, University of Pisa, Pisa, Italy; 6 School of Biosciences and Veterinary Medicine, University of Camerino, Camerino, Italy; 7 Department of Pharmacy, University of Pisa, Pisa, Italy; 8 School of Pharmacy, University of Camerino, Camerino, Italy

**Keywords:** Claustrum, Insula, Pig, Proteomic, Putamen

## Abstract

The present study, employing a comparative proteomic approach, analyzes the protein profile of pig claustrum (CLA), putamen (PU), and insula (IN). Pig brain is an interesting model whose key translational features are its similarities with cortical and subcortical structures of human brain. A greater difference in protein spot expression was observed in CLA vs PU as compared to CLA vs IN. The deregulated proteins identified in CLA resulted to be deeply implicated in neurodegenerative (i.e., sirtuin 2, protein disulfide-isomerase 3, transketolase) and psychiatric (i.e., copine 3 and myelin basic protein) disorders in humans. Metascape analysis of differentially expressed proteins in CLA vs PU comparison suggested activation of the α-synuclein pathway and L1 recycling pathway corroborating the involvement of these anatomical structures in neurodegenerative diseases. The expression of calcium/calmodulin-dependent protein kinase and dihydropyrimidinase like 2, which are linked to these pathways, was validated using western blot analysis. Moreover, the protein data set of CLA vs PU comparison was analyzed by Ingenuity Pathways Analysis to obtain a prediction of most significant canonical pathways, upstream regulators, human diseases, and biological functions. Interestingly, inhibition of presenilin 1 (PSEN1) upstream regulator and activation of endocannabinoid neuronal synapse pathway were observed. In conclusion, this is the first study presenting an extensive proteomic analysis of pig CLA in comparison with adjacent areas, IN and PUT. These results reinforce the common origin of CLA and IN and suggest an interesting involvement of CLA in endocannabinoid circuitry, neurodegenerative, and psychiatric disorders in humans.

**Supplementary Information:**

The online version contains supplementary material available at 10.1007/s12035-023-03347-2.

## Introduction

Claustrum (CLA) is a thin sheet of gray matter located in the forebrain between the insula (IN) and the putamen (PU). Although its precise role remains a matter of debate, CLA is thought to be implicated in a variety of functions such as attention [1–3], impulsivity [4], regulation of sleep [5–7], and consciousness [8]. Moreover, a recent new hypothesis indicates CLA as a possible limbic–sensory–motor interface [9]. The pivotal role of CLA in these functions is supported by its extensive, reciprocal connectivity with the entire neocortex [10–13].

Recently, a wealth of data has been accumulated on the role of CLA in different neurological disorders. Changes in CLA morphology are described in Parkinson’s disease (PD), Alzheimer’s disease (AD), autism, schizophrenia, and depressive disorders. However, there is a lack of information regarding the involvement of CLA in these disorders, at molecular level [14].

CLA origin is another puzzling problem, and morphogenetic and neurochemical similarities led some authors to postulate a common origin for CLA and the insular cortex [15, 16]; on the other hand, a subcortical origin has also been reported [17–19]. Furthermore, according to the hybrid ontogeny theory, CLA is considered as an intermediary between the cortical plate and corpus striatum [17].

Advances on the knowledge of CLA role in different species have essentially been obtained using immunohistochemical, physiological, and behavioral methods [1, 3, 10–13, 20–23]; a different approach, employing a proteomic analysis, has been used to establish the anatomical definitions of rat CLA [24]. Recently, a single-cell integrating transcriptomic and circuit-level approach, advanced by Erwin et al. [25], identified two excitatory CLA neuron subtypes that are molecularly distinguishable from the adjacent cortex.

The position of this structure, encased as it is between the external and extreme capsule, renders measurements, characterizations, and manipulations difficult; this is particularly true for rodent CLA where the extreme capsule is not clearly defined [26]. Among mammals, the pig brain is an interesting model whose key translational features are its similarities with cortical and subcortical structures of human brain [27, 28].

Furthermore, the most caudal part of pig CLA is characterized by a wide enlargement which is well delineated and separated from the adjoining structures [29], and this allows for isolation and sampling of CLA, IN, and PU without mixing tissues from different structures.

Here, we used a proteomic approach to define the protein profile of pig CLA and compare it with those of IN and PU. We then sought to reveal specific molecular hallmarks of pig CLA to better understand its function and origin, as well as possible implications of our findings in relation to human neurological diseases.

## Materials and Methods

### Animals and Tissue Samples

**Fig. 1:**
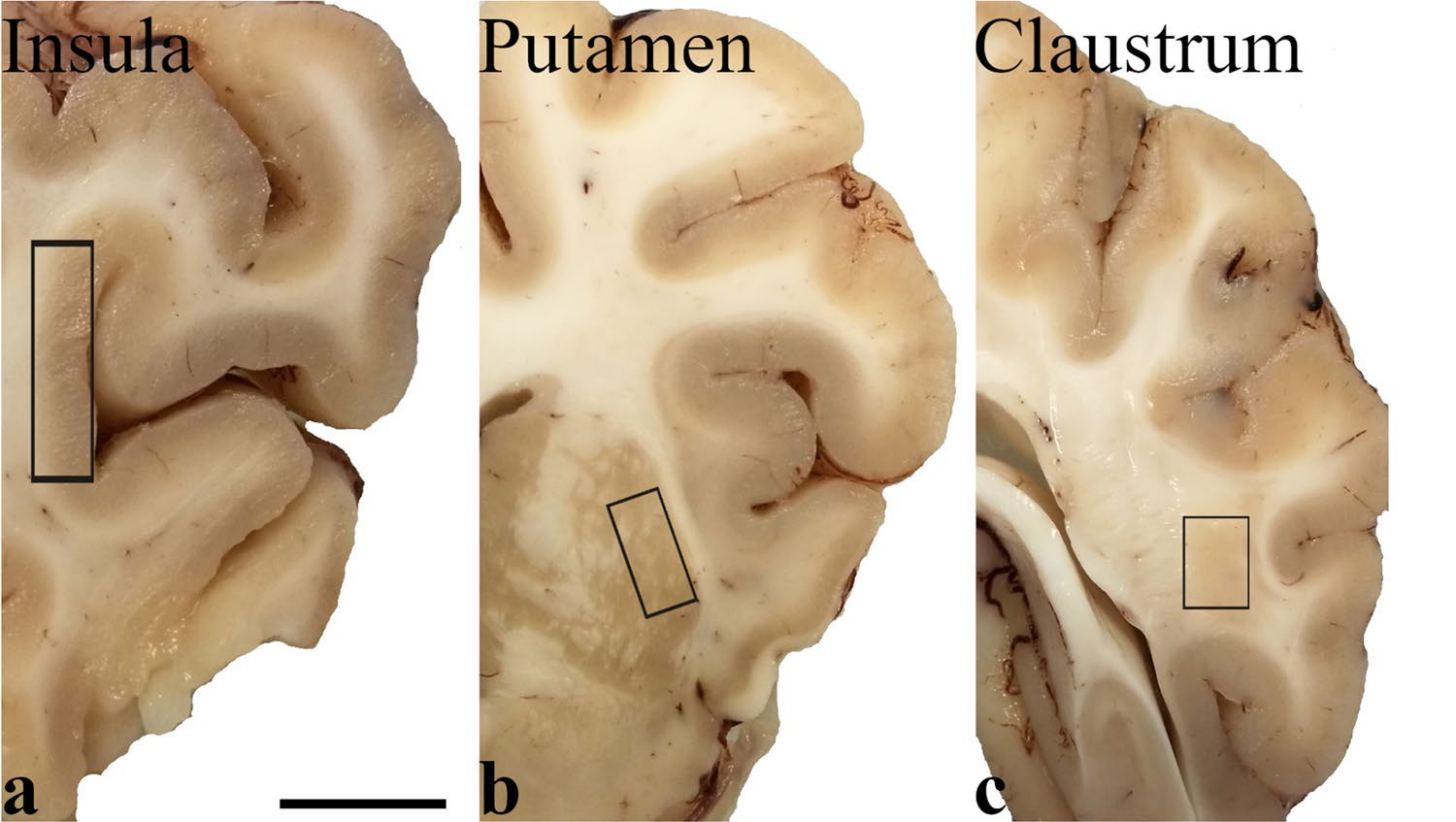
Photographs of coronal sections (**a** most cranial, **c** most caudal) of pig brain showing the sampled regions (black rectangles). Samples were taken from the insula (**a**), putamen (**b**), and caudal claustrum (**c**), and the latter was characterized by a large mass of about 0.5 cm in diameter. Scale bar = 1 cm

The brains of eight adult pigs (*Sus scrofa domesticus*) were removed immediately after commercial slaughtering at a local abattoir (Desideri Luciano SPA, Via Abruzzi, 2, 56025 Pontedera PI, Tuscany, Italy). Animals were treated according to the European Regulation (CE1099/2009) concerning animal welfare during the commercial slaughtering process and were constantly monitored under mandatory official veterinary medical care. All the animals were in good body conditions and considered free of pathologies by the veterinary medical officer responsible for the health and hygiene of the slaughterhouse. The brains, extracted within 15 min of death, were cut into transverse blocks (0.5 cm thick) containing CLA, PU, and IN in their rostro-caudal extent. Tissues of the right hemisphere were fixed by immersion in 4% paraformaldehyde in 0.1 M phosphate-buffered saline (PBS) at pH 7.4 and processed for paraffin embedding. From the left hemisphere, specimens of CLA, PU, and IN (Fig. 1) were quickly extracted under a stereomicroscope, snap-frozen in liquid nitrogen, and stored at − 80 °C until use.

### Protein Extractions and Proteomic Analysis

Specimens of CLA (*n* = 3), IN (*n* = 3), and PU (*n* = 3) were weighted, minced with scissors, and homogenized in 5 vol (w/V) of rehydration solution (7 M urea, 2 M thiourea, 4% 3-((3-cholamidopropyl) dimethylammonio)-1-propanesulfonate (CHAPS), 60 mM dithiothreitol (DTT) containing protease inhibitors (Merck KGaA, Darmstadt, Germany)) using a Teflon-glass homogenizer (10 strokes). The resulting homogenates were incubated for 1 h at room temperature (RT) with occasional stirring. Thereafter, samples were centrifuged at 17,000 *g* for 15 min at RT to eliminate insoluble materials. The protein content was measured by the RC/DC assay (Bio-Rad, Hercules, CA, USA) using bovine serum albumin as standard.

Two-dimensional electrophoresis (2-DE) was performed essentially according to Ciregia et al. [30]. Briefly, isoelectrofocusing (IEF) was carried out using a pH 3–10 nonlinear (NL) gradient. Two hundred μg of proteins was filled up to 400 μL in rehydration solution containing 1% IPG buffer at pH 3–10 NL and 0.8% Pharmalyte. Immobiline DryStrip gels were rehydrated overnight in the sample and then transferred to the Ettan IPGphor II (GE Healthcare Europe, Uppsala, Sweden) apparatus. The second dimension (SDS-PAGE) was carried out by transferring the proteins to 12% polyacrylamide gels, and at the end of the second dimension, gels were stained with 1 μM bathophenanthroline disulfonate-bis(2,2′-bipyridine)[Ru(II)] tetrahydrate (RuBP) (Cyanagen Srl, Bo, Italy) staining [31]. Images were acquired using ImageQuant LAS 4010 (GE HealthCare) and analyzed by SameSpots (V4.1, TotalLab, Newcastle Upon Tyne, UK) software which generates 2-DE analyses which are robust and accurate. Briefly, the gels were aligned to place all spots in exactly the same location, and then, the spot detection produced a complete data set since all gels contain the same number of spots, each matched to its corresponding spot on all gels. After 2-DE gel alignment and subsequent spot detection, the software calculated background-corrected abundance, by determining the lowest intensity value of the image pixels outside [30, 31].

### In-Gel Digestion and Mass Spectrometry

The gel pieces were digested as reported by Giusti et al. [32]. Samples were analyzed by LC-MS/MS as previously described [33] using a Proxeon EASY-nLC II (Thermo Fisher Scientific, Milan, Italy) chromatographic system coupled to a maXis HD UHR-TOF (Bruker Daltonics GmbH, Bremen, Germany) mass spectrometer. Briefly, peptides were loaded on the EASY-Column C18 trapping column (2 cm L, 100 μm ID, 5 μm ps; Thermo Fisher Scientific), and then separated on an Acclaim PepMap 100 C18 (25 cm L, 75 μm ID, 5 μm ps; Thermo Fisher Scientific) nanoscale chromatographic column at a flow rate of 300 nL/min and with a standard gradient from 3 to 35% of acetonitrile in 15 min. The mass spectrometer was equipped with a nanoESI spray source and operated in positive ion polarity and auto MS/MS mode (data-dependent acquisition (DDA)), using N_2_ as collision gas for collision-induced dissociation (CID) fragmentation. In-source reference lock mass (1221.9906 m/z) was acquired online throughout the runs.

Raw data were processed with DataAnalysis v. 4.2 to apply the lock mass calibration and then loaded in PEAKS Studio v7.5 software (Bioinformatic Solutions, Inc., Waterloo, Canada) using the “correct precursor only” option. The mass lists were searched against the NeXtProt database *Sus scrofa domesticus* (downloaded December 2018 and containing 42,184 entries). Carbamidomethylation of cysteines was selected as fixed modification, and oxidation of methionines, deamidation of asparagine and glutamine, as well as N terminus and lysine acetylation were set as variable modifications. Nonspecific cleavage was allowed to the one end of the peptides, with a maximum of 2 missed cleavages and 2 variable post-translational modifications (PTMs) per peptide; 10 ppm and 0.05 Da were set as the highest error mass tolerances for precursors and fragments, respectively; − 10logP threshold for peptide-spectrum matches (PSMs) was manually set from 15 to 35, in order to obtain a false discovery rate (FDR) value < 0.1% for both PSM and peptide sequences. For protein ID, the FDR value was < 0.1%.

### Western Blot Analysis

Aliquots (5 μg of proteins) of protein extracts from different brain regions (CLA, *n* = 5; IN, *n* = 5; PU, *n* = 5) were mixed with Laemmli solution, resolved on 4–16% polyacrylamide gels (Mini-PROTEAN® Precast Gels, Bio-Rad, Hercules, CA, USA) using a Mini-PROTEAN Tetra Cell (Bio-Rad), and transferred onto 0.2-μm nitrocellulose membranes using a Trans-Blot Turbo transfer system (Bio-Rad) (Ciregia et al., 2013). Membranes were blocked in TBST (50 mM Tris [pH 7.5], 150 mM NaCl, and 0.1% Tween 20), supplemented with 3% non-fat dry milk for 1 h at room temperature, and subsequently probed with the following primary antibodies: a mouse monoclonal anti-calcium/calmodulin-dependent protein kinase II-α (CaMKII-α, dilution 1:1000, 6G9; Cell Signaling Technology, Inc., Danvers, MA, USA) and a rabbit monoclonal anti-dihydropyrimidinase like 2 (DPYL2, alias collapsin response mediator protein 2 (CRMP-2), D8L6V, dilution 1:1000; Cell Signaling Technology, Inc., Danvers, MA, USA) in TBST/blocking solution overnight at 4 °C. Membranes were then incubated with the secondary antibody for 1 h at room temperature: HRP-goat anti-rabbit (Enzo Life Sciences, Inc., NY, USA) and HRP-goat anti-mouse (PerkinElmer, Inc., MA, USA) secondary antibodies were used at 1:10,000 dilution. Immunoblots were developed using the enhanced chemiluminescence (ECL) detection system, the chemiluminescent images were acquired using LAS 4010 (GE HealthCare), and the immunoreactive specific bands were quantified using ImageQuant L software. To normalize the optical density (OD) of immunoreactive bands, the OD of whole proteins was measured and, immediately after the electroblot, membranes were stained with 1 μM RuBPS [34]. Differences of protein expression levels among different samples were assessed using a paired Student’s *t* test (*p* < 0.05).

### Immunofluorescence

Immunofluorescence was performed on serial 5-μm sections using a mouse monoclonal anti-CaMKII-α (dilution 1:2000, 6G9; Cell Signaling Technology, Inc., Danvers, MA, USA) or a rabbit monoclonal anti-collapsin response mediator protein 2 (DPYL2 alias CRMP-2, dilution 1:200, D8L6V; Cell Signaling Technology, Inc., Danvers, MA, USA). Epitope retrieval was carried out at 120 °C in a pressure cooker for 5 min with a Tris/EDTA buffer, pH 9.0. Sections were blocked for 1 h with 5% normal horse serum (PK-7200, Vector Labs) in PBS and then incubated overnight at 4 °C in a solution of anti-CaMKII-α or anti-CRMP-2 in PBS containing 2% normal horse serum and 0.05% Triton X-100. Sections were then rinsed in PBS (3 × 10 min), incubated for 1 h at room temperature with DyLight 488 anti-mouse IgG (5 μg/mL, DI-2488; Vector Labs., Burlingame, CA, USA) or anti-rabbit IgG (5 μg/mL, DI-1088; Vector Labs., Burlingame, CA, USA). Finally, sections were washed with PBS and coverslipped with Vectashield medium containing 4′,6-diamidino-2-phenylindole (DAPI) (H-1500, Vector Labs). The specificity of immunohistochemical staining was tested using negative control sections, in which the primary or secondary antibody was replaced with PBS or non-immune serum. Under these conditions, nonspecific staining was absent.

Microphotographs were collected under a Nikon Ni-E light microscope (Nikon Instruments, Spa Calenzano, Florence, Italy), fully equipped for fluorescence acquisition, connected to a personal computer via Nikon digital image processing software (Digital Sight DS-U1, NIS-Elements BR 4.51.00 software). CLA and adjoining structures were identified according to a stereotaxic atlas [35].

### Statistical Analysis and Bioinformatics

All experiments were performed at least in triplicate, and resulting values are expressed as mean ± standard error.

In 2-DE experiments, a comparison among the different brain areas was performed, and the significance of the differences of normalized volume for each spot was calculated by the software SameSpots including the analysis of variance (ANOVA) test. Therefore, the protein spots that exhibited ratio ≥ 1.2 or ≤ 0.83, *p* value ≤ 0.05, and *q* value ≤ 0.05 were taken into consideration for further identification by nanoLC-MS/MS. Volcano plot and statistical analysis on individual proteins was performed using GraphPad Prism 8 (GraphPad Software, Inc., La Jolla, CA, USA). In western blot analysis, paired Student’s *t* test was used to compare differences among different brain areas, and differences with a *p* value < 0.05 were considered statistically significant.

The list of genes obtained from proteins found differentially expressed in different comparisons was analyzed using Metascape [36]. Metascape utilizes the well-adopted hypergeometric test [37] and the Benjamini–Hochberg *p* value correction algorithm [38] to identify all ontology terms that contain a statistically greater number of genes in common with an input list than expected by chance. Metascape automatically clusters enriched terms into non-redundant groups and chooses the most significant (lowest *p* value) term within each cluster to represent the cluster in heat map representations. Moreover, given a list of proteins, it automatically extracts a protein interaction network formed by these candidates. Finally, a circos plot showing how genes from the input gene lists overlap among different brain areas was generated.

Proteins found differentially expressed in each comparison were functionally analyzed using the Ingenuity Pathways Analysis (IPA; Qiagen, Redwood City, USA; www.qiagen.com/ingenuity, Build version: 321501M, Content version: 21249400) with the aim to determine the predominant functional relationships among proteins based on known associations in the literature. A comparison of the different analyses was created, and the upstream regulators, molecular functions, and human diseases whose activity appears to change in a significant manner according to the activation *z*-score value were shown. Heat map was build using NG-CHM GUI 2.20.2 software [39].

## Results

### Proteomic Analysis

**Fig. 2:**
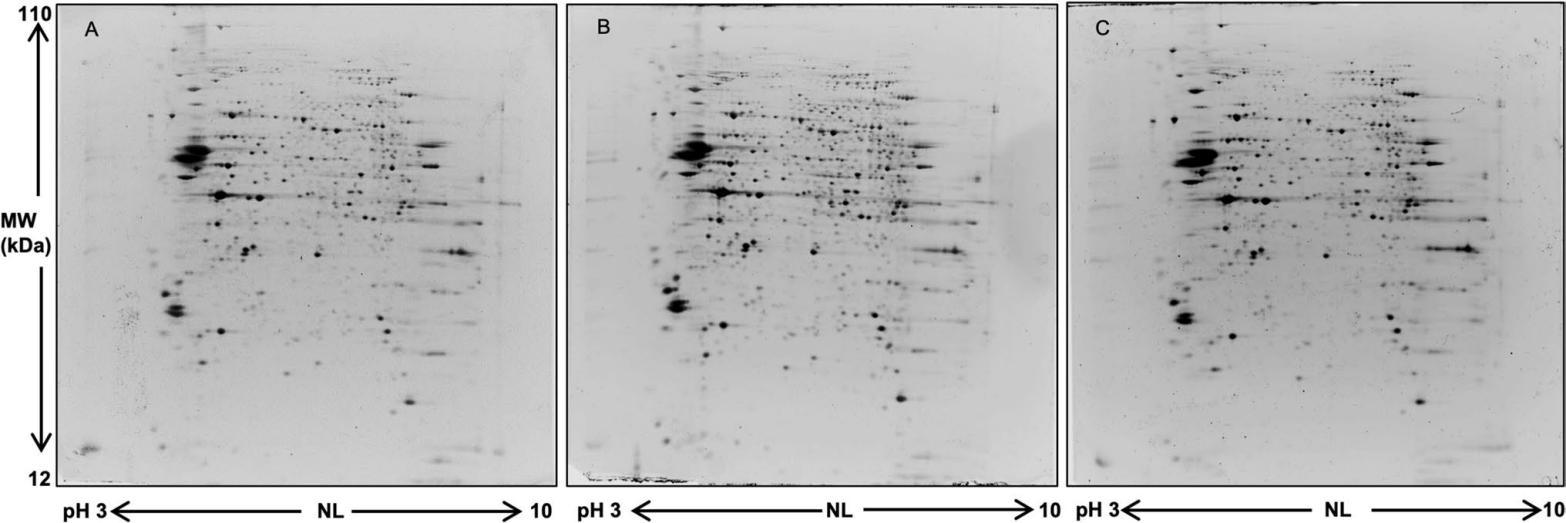
Representative 2-DE images of CLA (**A**), IN (**B**), and PU (**C**) protein profiles. Protein extracts were separated in a 3–10 nonlinear gradient. SDS-PAGE was performed using 12% acrylamide. Gels were stained with fluorescent dye and acquired by ImageQuant L

**Fig. 3:**
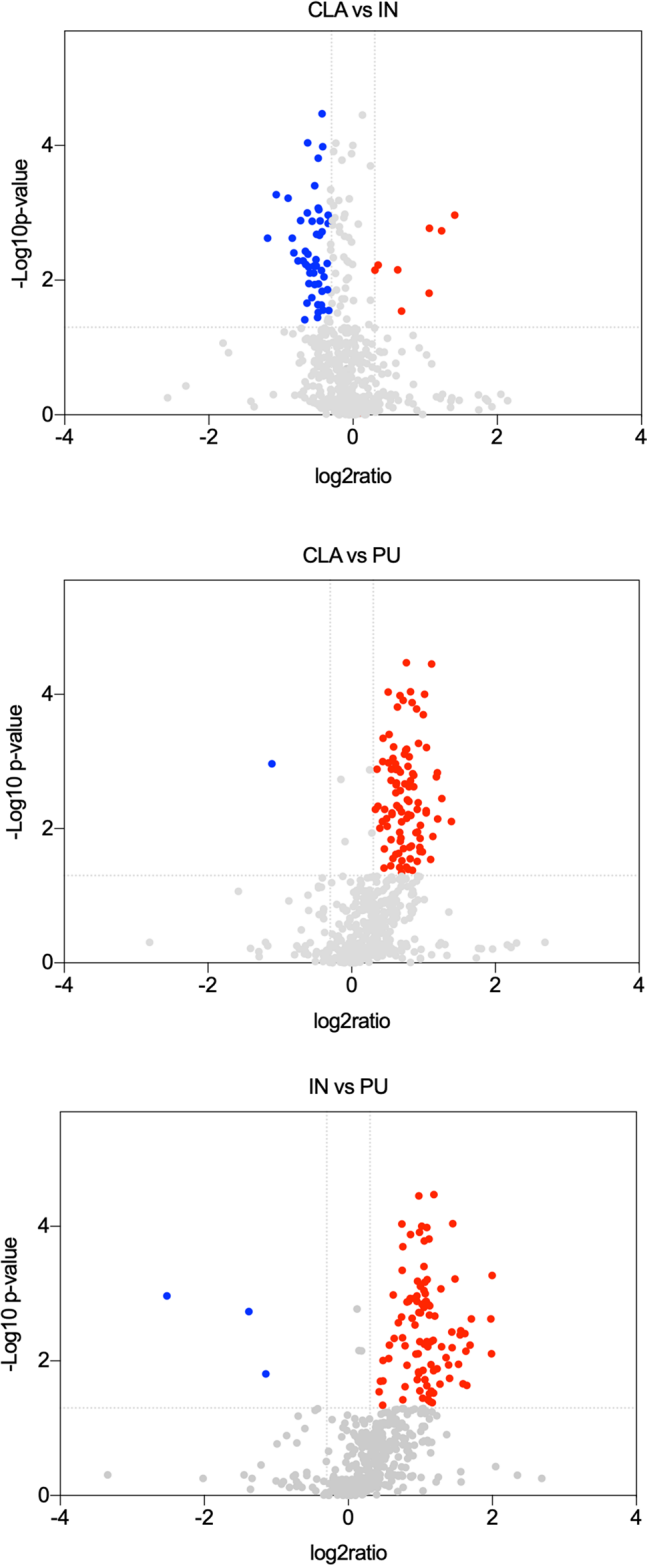
Scatter plot of fold change (*x*-axis) against log *p* value (*y*-axis) of all quantified proteins. Upregulated and downregulated proteins are colored red and blue, respectively. Dotted lines indicate the threshold of significance (*Y*-intercept) and ratio > 1.2 (*X*-intercept)

Two-dimensional electrophoresis was carried out to compare protein maps of different brain areas, and representative images of protein profile of CLA, IN, and PU are shown in Fig. 2A–C, respectively. Three different comparisons were performed: CLA vs IN, CLA vs PU, and IN vs PU. A greater difference of protein expression was observed between PU and the other two brain areas; in particular, 88 and 105 differentially expressed protein spots resulted from CLA vs PU and IN vs PU comparisons, respectively. On the contrary, minor significant differences in protein expression were observed in the comparison CLA vs IN. Volcano plots were constructed to graphically represent fold changes of protein expression (Fig. 3).

**Fig. 4:**
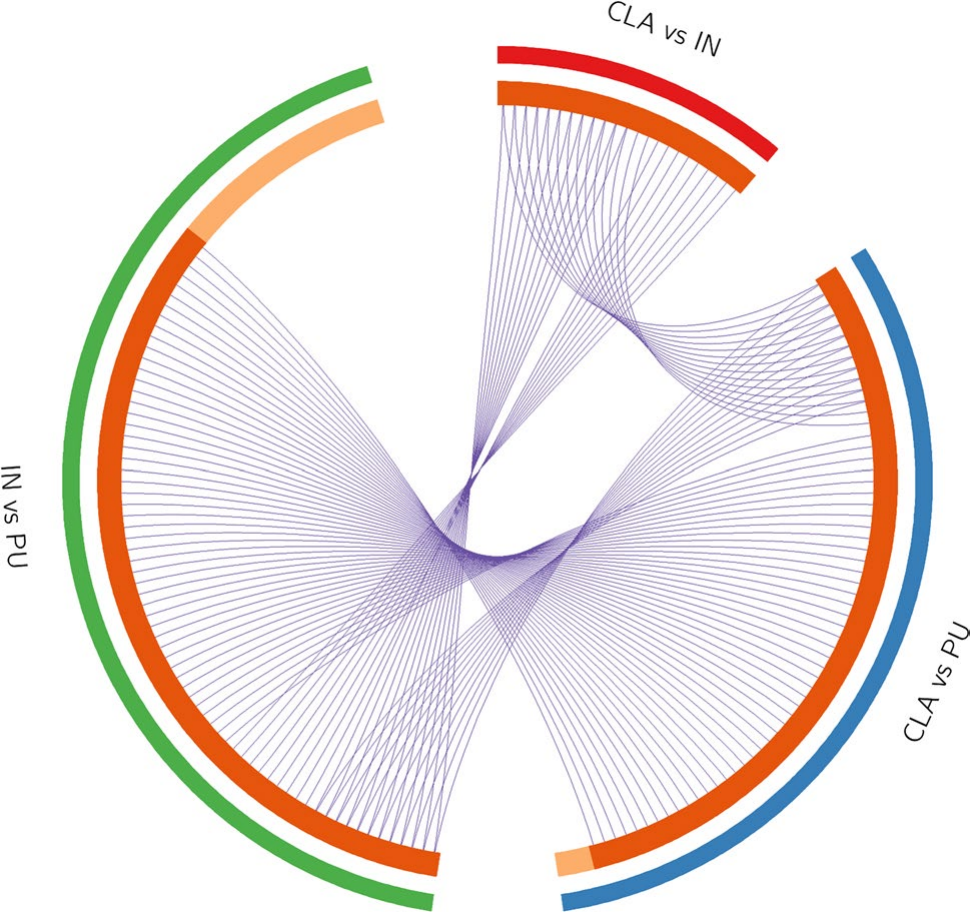
Circos plot showing how genes from the input gene lists overlap. On the outside, each arc represents the identity of each gene list: red (CLA vs IN), green (IN vs PU), and blue (CLA vs PU). On the inside, each arc represents a gene list, where each gene has a spot on the arc. The dark orange color represents the genes that appear in multiple lists, and the light orange color represents genes that are unique to that gene list. Purple lines link the same genes that are shared by multiple gene lists. More purple links and longer dark orange arcs imply a greater overlap among the input gene lists. CLA, claustrum; IN, insula; PU, putamen. Circos plot was generated using Metascape

**Fig. 5:**
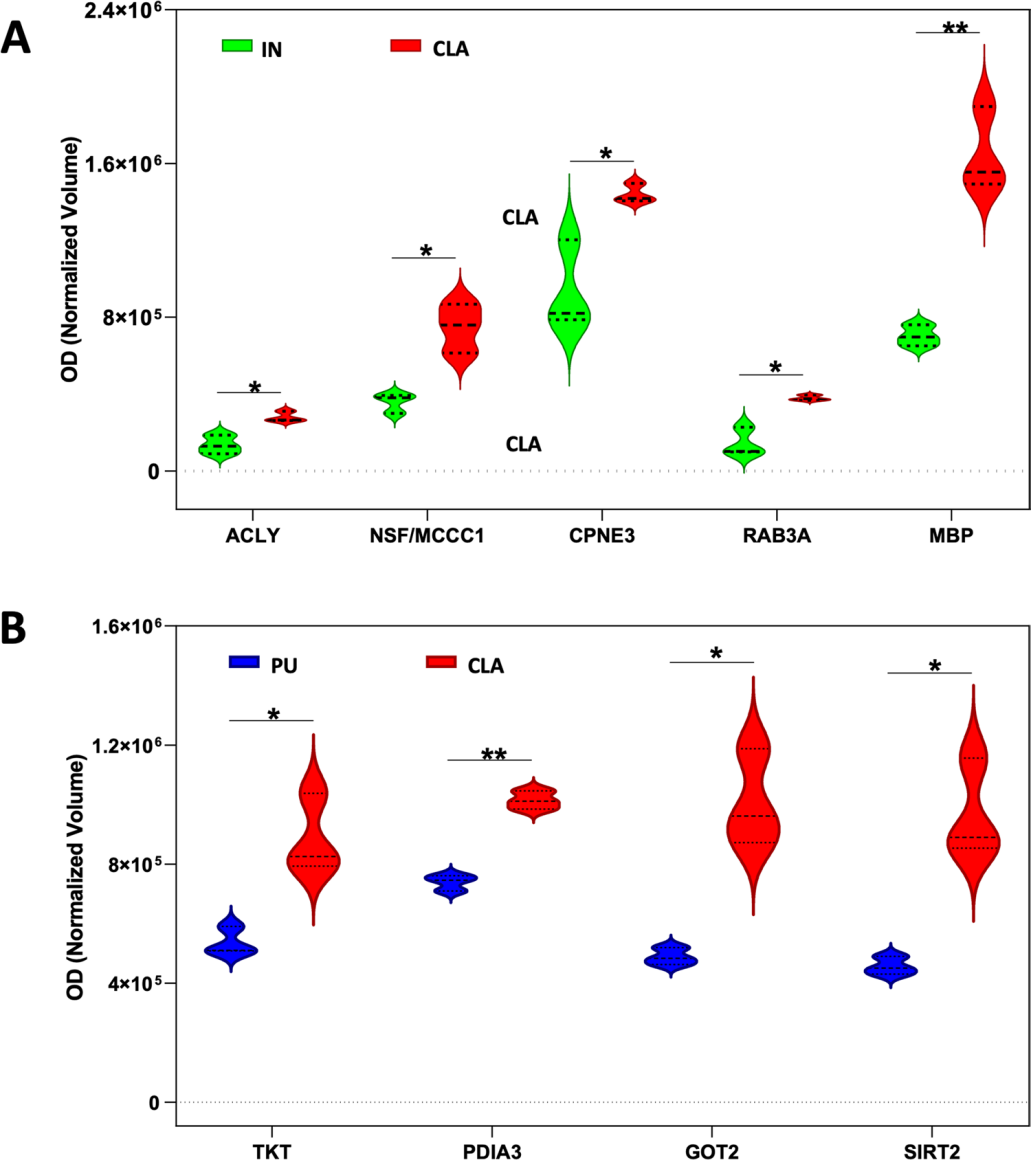
Violin plots of optical density (OD) normalized volumes of overexpressed proteins in claustrum (CLA) compared to insula (IN) (panel **A**) and putamen (PU) (panel **B**). Dashed lines represent the median whereas the dot lines represent the first and the third quartiles. Statistical analysis was performed using the Holm–Sidak method (*p < 0.05; **p < 0.01)

In Fig. 4, the circos plot shows how genes from the input gene lists obtained from different protein profile comparisons overlapped, and the protein divergence of CLA and IN when compared to PU placed both CLA and IN apart from PU with IN being the farthest. All spots showing an increase or decrease value ≥ 1.2 were subjected to nanoLC-ESI-MS/MS analysis and identified. Tables 1, 2, and 3 show the list of identified proteins together with their MW, pI, peptides and coverage values of MS/MS, ratio, and *p* values in three different comparisons. Twenty-one protein spots were identified in the CLA vs IN comparison of which only six proteins, Ras-related protein Rab-3A (RAB3A), ATP-citrate synthase (ACLY), methylcrotonyl-CoA carboxylase 1 (MCCC1), vesicle-fusing ATPase (NSF), copine 3 (CPN3), and myelin basic protein (MBP), resulted to be overexpressed in the CLA (Fig. 5).

**Table 1:** List of differentially expressed proteins obtained from the comparison CLA vs PU

Spot #	ID	Protein name	Gene	Coverage	Peptides	Unic	MW	pI	ANOVA *p* value	Ratio CLA/PU
364	F1RST0	HSPH1 heat shock protein 70 family	HSPH1	28	25	25	96,699	5.29	0.002	1.6
397	F1SUF2	Hexokinase	HK1	36	33	24	83,569	6.96	0.016	1.4
407	F1RWX8	Ubiquitin-like modifier-activating enzyme 1	UBA1	15	16	16	117,757	5.55	0.00004	1.7
415	F1SDW6	Oxoglutarate dehydrogenase like	OGDHL	26	28	21	115,227	6.39	0.026	1.7
471	F1RRW8	Dynamin 1	DNM1	39	43	10	97,328	7.97	0.037	1.9
476	F1RRW8	Dynamin 1	DNM1	39	38	11	97,328	7.97	0.012	1.5
490	F1SIH8	Transitional endoplasmic reticulum ATPase	VCP	46	45	44	89,431	5.44	0.00004	1.4
505	F1SFG7	Dynamin-like GTPase, mitochondrial	OPA1	33	25	25	78,120	8.07	0.034	1.9
637	F1RRS3	Vesicle-fusing ATPase	NSF	7	5	5	83,585	6.52	0.007	1.7
655	F1SII4	Glycyl-tRNA synthetase	GARS	19	18	18	83,260	7.02	0.02	1.9
665	F1RRS3	Vesicle-fusing ATPase	NSF	10	7	7	83,500	6.52	0.004	2.3
665	I3LT90	Methylcrotonyl-CoA carboxylase 1	MCCC1	3	2	2	80,400	6.34	0.004	2.3
673	F1RRS3	Vesicle-fusing ATPase	NSF	16	12	12	85,585	6.52	0.011	2
681	I3LJE2	Dihydropyrimidinase like 2	DPYSL2	20	14	14	73,531	5.94	0.014	1.6
684	I3LJE2	Dihydropyrimidinase like 2	DPYSL2	40	25	23	73,531	5.94	0.008	1.9
690	I3LJE2	Dihydropyrimidinase like 2	DPYSL2	20	14	14	73,531	5.94	0.004	2.5
751	C3RZ98	Protein arginine *N*-methyltransferase 5	PRMT5	8	6	6	72,614	5.88	0.005	1.8
765	F1RS11	Syntaxin-binding protein 1	STXBP1	30	23	23	68,749	6.32	0.031	1.3
777	A8U4R4	Transketolase	tkt	24	20	20	67,838	7.21	0.007	1.7
817	I3LJE2	Dihydropyrimidinase like 2	DPYSL2	37	27	26	73,531	5.94	0.003	1.7
819	F1RS11	Syntaxin-binding protein 1	STXBP1	31	24	24	68,749	6.32	0.026	1.5
820	I3LBY0	Coatomer subunit delta	ARCN1	14	7	7	57,250	5.69	0.008	1.6
824	I3LNG8	Stress-induced phosphoprotein 1	STIP1	25	17	17	47,879	6.36	0.00053	1.6
824	I3LJE2	Dihydropyrimidinase like 2	DPYSL2	9	6	6	73,531	5.94	0.00053	1.6
826	I3LJE2	Dihydropyrimidinase like 2	DPYSL2	37	30	23	73,531	5.94	0.01	1.5
827	I3LJE2	Dihydropyrimidinase like 2	DPYSL2	45	27	23	73,531	5.94	0.04	1.8
831	I3LJE2	Dihydropyrimidinase like 2	DPYSL2	44	57	53	73,531	5.94	0.00005	1.9
858	I3LJE2	Dihydropyrimidinase like 2	DPYSL2	11	4	3	73,531	5.94	0.007	1.9
878	I3LN38	Collapsin response mediator protein 1	CRMP-1	26	13	11	58,144	6.47	0.025	1.6
878	F1RGA9	Coronin	CORO1C	12	6	6	58,929	6.48	0.025	1.6
884	I3LR32	CCT-epsilon	CCT5	33	23	23	54,518	5.57	0.002	1.4
901	I3LDA5	EH domain containing 4	EHD4	11	7	3	42,252	6.32	0.002	2.4
918	F1RXD5	Copine 3	CPNE3	9	5	5	59,644	5.57	0.00011	1.7
933	F6QA08	Protein disulfide-isomerase	PDIA3	45	28	28	56,859	5.93	0.00031	1.4
940	I3LR17	Coronin 1A	CORO1A	27	16	16	43,411	6.12	0.008	2.3
964	F1RR02	Glial fibrillary acidic protein	GFAP	47	26	26	49,437	5.65	0.00001	1.8
964	F1RMZ8	ATPase H+ transporting V1 subunit B2	ATP6V1B2	17	9	9	56,613	5.57	0.00001	1.8
971	I3LGA1	WD repeat domain 37	WDR37	11	5	5	54,116	6.95	0.032	1.8
1007	D0G0C8	Chaperonin containing TCP1, subunit 2 (beta)	CCT2	27	16	16	57,444	6.09	0.036	1.8
1012	F1ST01	Selenium-binding protein 1	SELENBP1	29	17	17	52,534	6.54	0.016	1.9
1012	I3LKF3	Fascin	FSCN1	26	14	14	53,234	8.19	0.016	1.9
1015	I3LKF3	Fascin	FSCN1	34	16	16	53,234	8.19	0.016	1.7
1015	F1ST01	Selenium-binding protein 1	SELENBP1	9	3	3	52,534	6.54	0.016	1.7
1017	F1ST01	Selenium-binding protein 1	SELENBP1	10	5	5	52,534	6.54	0.021	1.9
1057	F1RR02	Glial fibrillary acidic protein	GFAP	55	37	36	49,437	5.65	0.00008	2.2
1062	F1RR02	Glial fibrillary acidic protein	GFAP	55	34	33	49,437	5.65	0.00029	2
1077	F1SEN2	Glutamate dehydrogenase 1, mitochondrial	GLUD1	19	10	10	61,308	8.02	0.014	1.5
1131	I3LNG5	Calcium/calmodulin-dependent protein kinase	CAMK2A	17	10	9	42,639	6.61	0.01	1.5
1135	I3LNG5	Calcium/calmodulin-dependent protein kinase	CAMK2A	17	10	10	42,639	6.61	0.01	1.7
1137	I3LNG5	Calcium/calmodulin-dependent protein kinase	CAMK2A	19	11	2	42,639	6.61	0.001	1.9
1146	I3LNG5	Calcium/calmodulin-dependent protein kinase	CAMK2A	19	11	2	42,639	6.61	0.026	1.8
1146	I3LK59	2-Phospho-*d*-glycerate hydro-lyase	ENO1	9	4	4	38,082		0.026	1.8
1153	I3LNG5	Calcium/calmodulin-dependent protein kinase	CAMK2A	23	12	2	42,639	6.61	0.013	1.5
1170	F1RUK8	Rab GDP dissociation inhibitor	GDI2	52	24	21	50,327	5.78	0.009	1.5
1187	F1RR48	SH3 domain containing GRB2 like, endophilin B2	SH3GLB2	13	6	6	43,489	5.73	0.00039	1.6
1194	Q29387	Elongation factor 1-gamma	EEF1G	31	21	21	49,624	6.16	0.019	2
1195	A6M928	Eukaryotic translation initiation factor 4A isoform 1	EIF4A1	22	9	3	46,154	5.32	0.002	2.8
1195	A6M930	Eukaryotic translation initiation factor 4A isoform 2	EIF4A2	21	9	3	46,502	5.33	0.002	2.8
1276	F1RK10	Succinate-CoA ligase (ADP-forming) subunit beta, mitochondrial	SUCLA2	31	18	18	50,304	5.86	0.029	1.3
1315	F1SNE5	SH3 domain containing GRB2 like 2, endophilin A1	SH3GL2	37	17	17	37,614	5.26	0.00048	2.3
1336	K7GM43	Septin 5	SEPT5	26	13	13	43,787	6.55	0.006	1.8
1363	P46410	Glutamine synthetase	GLUL	9	5	5	42,030	6.28	0.001	1.7
1376	F1RZB5	Tropomodulin 2	TMOD2	24	12	12	39,693	5.17	0.009	1.6
1414	F1RJ25	Fructose-bisphosphate aldolase	ALDOC	47	25	25	39,377	6.21	0.02	1.6
1421	F1RL02	Mitogen-activated protein kinase	MAPK1	39	15	14	37,974	6.56	0.042	1.6
1455	F1RF18	G protein subunit alpha o1	GNAO1	13	6	4	40,078	5.10	0.00037	1.4
1457	P00506	Aspartate aminotransferase, mitochondrial	GOT2	10	6	6	47,436	9.14	0.002	2.1
1464	Q6QAQ1	Actin, cytoplasmic 1	ACTB	11	6	6	41,737	5.29	0.02	1.5
1479	Q9TV69	Trans-1,2-dihydrobenzene-1,2-diol dehydrogenase	DHDH	27	10	10	36,527	6.34	0.003	1.6
1508	I3L8A1	NAD-dependent protein deacetylase	SIRT2	20	10	9	35,964	7.47	0.001	2.1
1520	F1RIK3	Acyl-CoA thioesterase 7	ACOT7	8	4	4	40,189	8.06	0.003	1.6
1524	F1SEX0	Dimethylarginine dimethylaminohydrolase 1	DDAH1	25	5	5	20,779	5.36	0.002	1.8
1524	F1SUE3	Pyrophosphatase (inorganic) 1	PPA1	10	3	3	32,790	5.44	0.002	1.8
1542	F1RPC8	Crystallin mu	CRYM	44	23	23	33,508	5.16	0.004	1.8
1580	P00336	l-Lactate dehydrogenase B chain	LDHB	37	18	18	36,612	5.57	0.00026	1.5
1592	F2Z4Z8	G protein subunit beta 2	GNB2	23	8	7	33,758	5.60	0.00082	1.4
1627	I3LSK5	G protein subunit beta 1	GNB1	35	13	13	37,331	5.60	0.00038	2
1654	F1RM45	Apolipoprotein E	APOE	20	7	7	36,665	5.92	0.006	2.1
1655	F1RM45	Apolipoprotein E	APOE	12	4	4	36,665	5.92	0.002	1.6
1660	F1SGH5	Pyruvate dehydrogenase E1 component subunit beta	PDHB	52	17	17	39,273	6.20	0.002	1.5
1660	F1RM45	Apolipoprotein E	APOE	22	6	6	36,665	5.92	0.002	1.5
1757	Q06A94	Heterogeneous nuclear ribonucleoprotein A1	HNRNPA1L2	28	8	8	34,196	9.27	0.003	2.2
1826	P62258	14-3-3 protein epsilon	YWHAE	69	38	38	29,174	4.63	0.007	1.4
2013	Q45FY6	Hypoxanthine-guanine phosphoribosyltransferase	HPRT1	57	14	14	24,555	6.30	0.014	2.6
2029	Q6SEG5	Ubiquitin carboxyl-terminal hydrolase isozyme L1	UCHL1	66	38	37	24,859	5.22	0.00053	1.5
2100	Q06AU3	Ras-related protein Rab-3A	RAB3A	15	3	3	24,970	4.85	0.006	0.45
2165	Q6SEG5	Ubiquitin carboxyl-terminal hydrolase isozyme L1	UCHL1	65	38	7	24,859	5.22	0.011	1.6
2165	Q5E946	Protein DJ-1	PARK7	56	16	16	20,035	6.84	0.011	*1.6*
2402	A8QW48	Beta-synuclein	SNCB	25	5	4	14,115	4.46	0.002	1.6
2467	Q3I5G7	Alpha-synuclein	SNCA	53	8	8	14,520	4.62	0.002	1.7
2497	Q8WNW3	Junction plakoglobin	Jup	27	20	20	81,850	5.75	0.0003	2.4

**Table 2:** List of differentially expressed proteins obtained from the comparison CLA vs IN

Spot #	ID	Protein name	Gene	Coverage	Peptides	Unic	MW	pI	ANOVA *p* value	Ratio CLA/IN
345	F1S0N2	ATP-citrate synthase	ACLY	21	11	11	57,558	7.12	0.025	2.1
397	F1SUF2	Hexokinase	HK1	36	33	24	83,569	6.96	0.01	0.69
546	F1SCS1	DEAD-box helicase 1	DDX1	14	10	10	77,025	6.80	0.013	0.588
665	F1RRS3	Vesicle-fusing ATPase	NSF	10	7	7	83,500	6.52	0.005	2.1
665	I3LT90	Methylcrotonyl-CoA carboxylase 1	MCCC1	3	2	2	80,400	6.34	0.005	2.1
918	F1RXD5	Copine 3	CPNE3	9	5	5	59,644	5.57	0.03	1.5
959	I3L7D3	Synapsin II	SYN2	31	18	16	51,020	8.84	0.004	0.625
959	Q19PY3	tRNA-splicing ligase RtcB homolog	RTCB	31	17	17	55,238	6.77	0.004	0.625
959	I3LK72	Acyl-CoA synthetase family member 3	ACSF3	27	13	13	48,212	8.71	0.004	0.625
959	F1SLF6	Chaperonin containing TCP1, subunit 7 (Eta)	CCT7	24	14	14	59,471	6.74	0.004	0.625
960	I3L7D3	Synapsin II	SYN2	12	6	6	51,020	8.84	0.045	0.625
960	F1SD97	Phenylalanyl-tRNA synthetase alpha subunit	FARSA	18	10	10	57,628	7.85	0.045	0.625
1131	I3LNG5	Calcium/calmodulin-dependent protein kinase	CAMK2A	17	10	9	42,639	6.61	0.015	0.526
1137	I3LNG5	Calcium/calmodulin-dependent protein kinase	CAMK2A	19	11	2	42,639	6.61	0.021	0.476
1143	I3LK59	2-Phospho-d-glycerate hydro-lyase	ENO1	34	13	10	38,082	6.43	0.029	0.435
1143	I3LNG5	Calcium/calmodulin-dependent protein kinase	CAMK2A	21	12	3	42,639	6.61	0.029	0.435
1154	I3LNG5	Calcium/calmodulin-dependent protein kinase	CAMK2A	29	15	4	42,639	6.61	0.008	0.664
1187	F1RR48	SH3 domain containing GRB2 like, endophilin B2	SH3GLB2	13	6	6	43,489	5.73	0.023	0.714
1464	Q6QAQ1	Actin, cytoplasmic 1	ACTB	11	6	6	41,737	5.29	0.034	0.714
1542	F1RPC8	Crystallin mu	CRYM	44	23	23	33,508	5.16	0.003	0.667
1592	F2Z4Z8	G protein subunit beta 2	GNB2	23	8	7	33,758	5.60	0.042	0.833
1815	Q9GZU5	Nyctalopin	NYX	2	2	2	52,000	9.10	0.026	0.588
1826	P62258	14-3-3 protein epsilon	YWHAE	69	38	38	29,174	4.63	0.044	0.833
2100	Q06AU3	Ras-related protein Rab-3A	RAB3A	15	3	3	24,970	4.85	0.018	2.7
2402	A8QW48	Beta-synuclein	SNCB	25	5	4	14,115	4.46	0.02	0.769
2467	Q3I5G7	Alpha-synuclein	SNCA	53	8	8	14,520	4.62	0.013	0.769
2487	P81558	Myelin basic protein	MBP	27	4	4	18,486	11.28	0.00061	2.3

**Table 3:** List of differentially expressed proteins obtained from the comparison IN vs PU

Spot #	ID	Protein name	Gene	Coverage	Peptides	Unic	MW	pI	ANOVA *p* value	Ratio IN/PU
332	F1SCV1	Gamma-tubulin complex component	TUBGCP2	1	2	1	102,582	6.26	0.031	2.1
345	F1S0N2	ATP-citrate synthase	ACLY	21	11	11	57,558	7.12	0.034	0.45
355	I3L8X6	Amphiphysin	AMPH	19	15	15	71,840	4.56	0.006	2.2
364	F1RST0	Heat shock 110 kDa protein	HSPH1	28	25	25	96,699	5.29	0.005	2
365	F1RST0	Heat shock 110 kDa protein	HSPH1	17	15	15	96,699	5.29	0.001	2
394	F1SML4	Staphylococcal nuclease and Tudor domain containing 1	SND1	8	5	4	66,087	6.72	0.038	2
397	F1SUF2	Hexokinase	HK1	36	33	24	83,569	6.96	0.00085	2.1
407	F1RWX8	Ubiquitin-like modifier-activating enzyme 1	UBA1	15	16	16	117,757	5.55	0.003	2.4
415	F1SDW6	Oxoglutarate dehydrogenase like	OGDHL	26	28	21	115,227	6.39	0.032	2.2
455	F1RI39	Actinin alpha 4	ACTN4	27	20	20	101,837	5.23	0.002	2.5
471	F1RRW8	Dynamin 1	DNM1	39	43	10	97,328	7.97	0.011	2.6
476	F1RRW8	Dynamin 1	DNM1	39	38	11	97,328	7.97	0.001	2.1
490	F1SIH8	Transitional endoplasmic reticulum ATPase	VCP	46	45	44	89,431	5.44	0.02	1.5
505	F1SFG7	OPA1, mitochondrial dynamin-like GTPase	OPA1	33	25	25	78,120	8.07	0.01	2.9
546	F1SCS1	DEAD-box helicase 1	DDX1	14	10	10	77,025	6.80	0.00034	2.1
630	I3L8C5	Heat shock protein family A (Hsp70) member 12A	HSPA12A	20	14	14	74,809	6.18	0.01	2
637	F1RRS3	Vesicle-fusing ATPase	NSF	7	5	5	83,585	6.52	0.007	2.7
655	F1SII4	Glycyl-tRNA synthetase	GARS	19	18	18	83,260	7.02	0.012	2.6
681	I3LJE2	Dihydropyrimidinase like 2	DPYSL2	20	14	14	73,531	5.94	0.005	2.3
684	I3LJE2	Dihydropyrimidinase like 2	DPYSL2	40	25	23	73,531	5.94	0.007	2.9
690	I3LJE2	Dihydropyrimidinase like 2	DPYSL2	20	14	14	73,531	5.94	0.002	4.4
720	F1RPU0	Glycerol-3-phosphate dehydrogenase	GPD2	38	34	34	80,921	6.54	0.022	2.1
724	P28491	Calreticulin	CALR	36	23	23	48,288	4.32	0.01	1.5
751	C3RZ98	Protein arginine *N*-methyltransferase 5	PRMT5	8	6	6	72,614	5.88	0.01	1.5
765	F1RS11	Syntaxin-binding protein 1	STXBP1	30	23	23	68,749	6.32	0.002	1.4
799	O75083	WD repeat-containing protein 1	WDR1	16	11	2	66,194	6.17	0.001	1.7
817	I3LJE2	Dihydropyrimidinase like 2	DPYSL2	37	27	26	73,531	5.94	0.002	1.9
819	F1RS11	Syntaxin-binding protein 1	STXBP1	31	24	24	68,749	6.32	0.002	1.9
820	I3LBY0	Coatomer subunit delta	ARCN1	14	7	7	57,250	5.69	0.028	2.1
825	I3LNG8	Stress-induced phosphoprotein 1	STIP1	35	21	21	47,879	6.36	0.038	2.1
825	I3LJE2	Dihydropyrimidinase like 2	DPYSL2	3	2	2	73,531	5.94	0.038	2.1
826	I3LJE2	Dihydropyrimidinase like 2	DPYSL2	37	30	23	73,531	5.94	0.029	1.7
827	I3LJE2	Dihydropyrimidinase like 2	DPYSL2	45	27	23	73,531	5.94	0.012	2.7
831	I3LJE2	Dihydropyrimidinase like 2	DPYSL2	44	57	53	73,531	5.94	0.001	2.1
858	I3LJE2	Dihydropyrimidinase like 2	DPYSL2	11	4	3	73,531	5.94	0.014	2
878	I3LN38	Collapsin response mediator protein 1	CRMP-1	26	13	11	58,144	6.47	0.01	2.2
878	F1RGA9	Coronin	CORO1C	12	6	6	58,929	6.48	0.01	2.2
884	I3LR32	CCT-epsilon	CCT5	33	23	23	54,518	5.57	0.004	1.5
901	I3LDA5	EH domain containing 4	EHD4	11	7	3	42,252	6.32	0.007	3
933	E1CAJ5	Protein disulfide-isomerase	PDIA3	45	28	28	56,859	5.93	0.049	1.4
940	I3LR17	Coronin 1A	CORO1A	27	16	16	43,411	6.12	0.01	3.1
959	I3L7D3	Synapsin II	SYN2	31	18	16	51,020	8.84	0.002	2.1
959	Q19PY3	tRNA-splicing ligase RtcB homolog	RTCB	31	17	17	55,238	6.77	0.002	2.1
959	I3LK72	Acyl-CoA synthetase family member 3	ACSF3	27	13	13	48,212	8.71	0.002	2.1
959	F1SLF6	Chaperonin containing TCP1, subunit 7 (Eta)	CCT7	24	14	14	59,471	6.74	0.002	2.1
960	I3L7D3	Synapsin II	SYN2	12	6	6	51,020	8.84	0.046	2
960	F1SD97	Phenylalanyl-tRNA synthetase alpha subunit	FARSA	18	10	10	57,628	7.85	0.046	2
964	F1RR02	Glial fibrillary acidic protein	GFAP	47	26	26	49,437	5.65	0.001	1.8
964	F1RMZ8	ATPase H+ transporting V1 subunit B2	ATP6V1B2	17	9	9	56,613	5.57	0.001	1.8
971	I3LGA1	WD repeat domain 37	WDR37	11	5	5	54,116	6.95	0.033	2.2
1007	D0G0C8	Chaperonin containing TCP1, subunit 2 (beta) OS = *Sus scrofa*	CCT2	27	16	16	57,444	6.09	0.036	2.2
1012	F1ST01	Selenium-binding protein 1 OS	SELENBP1	29	17	17	52,534	6.54	0.034	2.1
1012	I3LKF3	Fascin	FSCN1	26	14	14	53,234	8.19	0.034	2.1
1015	I3LKF3	Fascin	FSCN1	34	16	16	53,234	8.19	0.024	1.9
1015	F1ST01	Selenium-binding protein 1	SELENBP1	9	3	3	52,534	6.54	0.024	1.9
1017	F1ST01	Selenium-binding protein 1	SELENBP1	10	5	5	52,534	6.54	0.012	2.3
1049	F1RR02	Glial fibrillary acidic protein	GFAP	43	23	23	49,437	5.65	0.003	2
1057	F1RR02	Glial fibrillary acidic protein	GFAP	55	37	36	49,437	5.65	0.00049	2
1062	F1RR02	Glial fibrillary acidic protein	GFAP	55	34	33	49,437	5.65	0.002	1.7
1064	F1SEN2	Glutamate dehydrogenase 1, mitochondrial	GLUD1	26	16	16	61,608	8.02	0.012	2.2
1064	F1RUE3	Succinate-semialdehyde dehydrogenase	ALDH5A1	11	6	6	57,784	8.61	0.012	2.2
1064	D2KPI8	Adenylosuccinate lyase	ADSL	7	4	4	55,092	6.45	0.012	2.2
1077	F1SEN2	Glutamate dehydrogenase 1, mitochondrial	GLUD1	19	10	10	61,308	8.02	0.001	1.8
1131	I3LNG5	Calcium/calmodulin-dependent protein kinase	CAMK2A	17	10	9	42,639	6.61	0.002	2.8
1137	I3LNG5	Calcium/calmodulin-dependent protein kinase	CAMK2A	19	11	2	42,639	6.61	0.002	4
1143	I3LK59	2-Phospho-d-glycerate hydro-lyase	ENO1	34	13	10	38,082	6.43	0.002	3.9
1143	I3LNG5	Calcium/calmodulin-dependent protein kinase	CAMK2A	21	12	3	42,639	6.61	0.002	3.9
1146	I3LNG5	Calcium/calmodulin-dependent protein kinase	CAMK2A	19	11	2	42,639	6.61	0.002	3.3
1146	I3LK59	2-Phospho-*d*-glycerate hydro-lyase	ENO1	9	4	4	38,082		0.002	3.3
1153	I3LNG5	Calcium/calmodulin-dependent protein kinase	CAMK2A	23	12	2	42,639	6.61	0.001	1.9
1154	I3LNG5	Calcium/calmodulin-dependent protein kinase	CAMK2A	29	15	4	42,639	6.61	0.003	1.8
1170	F1RUK8	Rab GDP dissociation inhibitor	GDI2	52	24	21	50,327	5.78	0.013	1.5
1187	F1RR48	SH3 domain containing GRB2 like, endophilin B2	SH3GLB2	13	6	6	43,489	5.73	0.00074	2.2
1194	Q29387	Elongation factor 1-gamma	EEF1G	31	21	21	49,624	6.16	0.024	2.4
1195	A6M928	Eukaryotic translation initiation factor 4A isoform 1	EIF4A1	22	9	3	46,154	5.32	0.004	2.1
1195	A6M930	Eukaryotic translation initiation factor 4A isoform 2	EIF4A2	21	9	3	46,502	5.33	0.004	2.1
1244	F2Z5G5	ARP1 actin-related protein 1 homolog A	ACTR1A	32	15	15	42,614	6.19	0.016	2.3
1244	F1RFI1	Elongation factor Tu	TUFM	18	9	8	49,451	6.72	0.016	2.3
1276	F1RK10	Succinate-CoA ligase (ADP-forming) subunit beta, mitochondrial	SUCLA2	31	18	18	50,304	5.86	0.007	1.6
1288	F1RHA0	2-Oxoisovalerate dehydrogenase subunit alpha	TMEM91	16	6	6	47,020	6.87	0.019	2
1315	F1SNE5	SH3 domain containing GRB2 like 2, endophilin A1	SH3GL2	37	17	17	37,614	5.26	0.008	2.2
1336	K7GM43	Septin 5	SEPT5	26	13	13	43,787	6.55	0.012	2.7
1363	P46410	Glutamine synthetase	GLUL	9	5	5	42,030	6.28	0.006	2.3
1376	F1RZB5	Tropomodulin 2	TMOD2	24	12	12	39,693	5.17	0.019	2
1409	F1SNE5	SH3 domain containing GRB2 like 2, endophilin A1	SH3GL2	19	11	11	37,614	5.26	0.00036	2.2
1413	F1S0R4	V-type proton ATPase subunit C	ATP6V1C1	27	14	14	44,033	7.62	0.002	2.1
1414	F1RJ25	Fructose-bisphosphate aldolase	ALDOC	47	25	25	39,377	6.21	0.00001	1.9
1421	F1RL02	Mitogen-activated protein kinase	MAPK1	39	15	14	37,974	6.56	0.00001	1.9
1464	Q6QAQ1	Actin, cytoplasmic 1	ACTB	11	6	6	41,737	5.29	0.0007	2.1
1469	F1RF18	G protein subunit alpha o1	GNAO1	8	5	5	40,078	5.10	0.00065	2
1479	Q9TV69	Trans-1,2-dihydrobenzene-1,2-diol dehydrogenase	DHDH	27	10	10	36,527	6.34	0.021	2.1
1487	F2Z5H6	V-type proton ATPase subunit	ATP6V0D1	23	10	10	40,329	4.89	0.003	2.2
1520	F1RIK3	Acyl-CoA thioesterase 7	ACOT7	8	4	4	40,189	8.06	0.011	2.1
1524	F1SEX0	Dimethylarginine dimethylaminohydrolase 1	DDAH1	25	5	5	20,779	5.36	0.005	1.8
1524	F1SUE3	Pyrophosphatase (inorganic) 1	PPA1	10	3	3	32,790	5.44	0.005	1.8
1542	F1RPC8	Crystallin mu	CRYM	44	23	23	33,508	5.16	0.00056	2.7
1569	F1RMB1	Phytanoyl-CoA 2-hydroxylase-interacting protein	PHYHIP	9	4	4	38,539	6.70	0.001	2
1580	P00336	l-Lactate dehydrogenase B chain	LDHB	37	18	18	36,612	5.57	0.005	1.8
1592	F2Z4Z8	G protein subunit beta 2	GNB2	23	8	7	33,758	5.60	0.00032	1.7
1614	K7GNZ3	NAC-A/B domain-containing protein	NACA	9	2	2	23,384	4.52	0.003	2
1627	I3LSK5	G protein subunit beta 1	GNB1	35	13	13	37,331	5.60	0.00007	2
1645	C5H0C6	Ubiquitin thioesterase	OTUB1	10	4	4	31,284	4.85		2.1
1654	F1RM45	Apolipoprotein E	APOE	20	7	7	36,665	5.92	0.009	2.1
1655	F1RM45	Apolipoprotein E	APOE	12	4	4	36,665	5.92	0.006	1.6
1660	F1SGH5	Pyruvate dehydrogenase E1 component subunit beta	PDHB	52	17	17	39,273	6.20	0.007	1.7
1660	F1RM45	Apolipoprotein E	APOE	22	6	6	36,665	5.92	0.007	1.7
1732	I3LRS8	Phosphatidylinositol transfer protein alpha	PITPNA	50	19	19	31,820	6.71	0.019	3
1757	Q06A94	Heterogeneous nuclear ribonucleoprotein A1	HNRNPA1L2	28	8	8	34,196	9.27	0.032	2.3
1815	Q9GZU5	NYX_HUMAN nyctalopin	NYX	2	2	2	52,000	9.10	0.008	2.1
1826	P62258	14-3-3 protein epsilon	YWHAE	69	38	38	29,174	4.63	0.00034	1.7
1939	F1S8Y5	Phosphoglycerate mutase	PGAM1	19	5	5	29,301	6.41	0.006	3.1
2013	Q45FY6	Hypoxanthine-guanine phosphoribosyltransferase	HPRT1	57	14	14	24,555	6.30	0.007	4
2029	Q6SEG5	Ubiquitin carboxyl-terminal hydrolase isozyme L1	UCHL1	66	38	37	24,859	5.22	0.012	1.7
2087	I3L9H4	PITH domain containing 1	PITHD1	27	5	5	24,265	5.47	0.006	2
2100	Q06AU3	Ras-related protein Rab-3A	RAB3A	15	3	3	24,970	4.85	0.004	0.175
2165	Q6SEG5	Ubiquitin carboxyl-terminal hydrolase isozyme L1	UCHL1	65	38	7	24,859	5.22	0.044	1.7
2165	Q5E946	Protein/nucleic acid deglycase DJ-1	PARK7	56	16	16	20,035	6.84	0.044	1.7
2402	A8QW48	Beta-synuclein	SNCB	25	5	4	14,115	4.46	0.00041	2.1
2467	Q3I5G7	Alpha-synuclein	SNCA	53	8	8	14,520	4.62	0.00003	2.3
2479	Q6DUB7	Stathmin	STMN1	46	8	8	17,302	5.75	0.011	3.2
2487	P81558	Myelin basic protein	MBP	27	4	4	18,486	11.28	0.006	0.38
2497	Q8WNW3	Junction plakoglobin	Jup	27	20	20	81,850	5.75	0.00076	3.2

On the contrary, in CLA vs PU comparison, an increase of expression was observed for whole differentially expressed proteins except RAB3A, which showed a higher expression level in PU compared to CLA and IN (Fig. 5B).

**Fig. 6:**
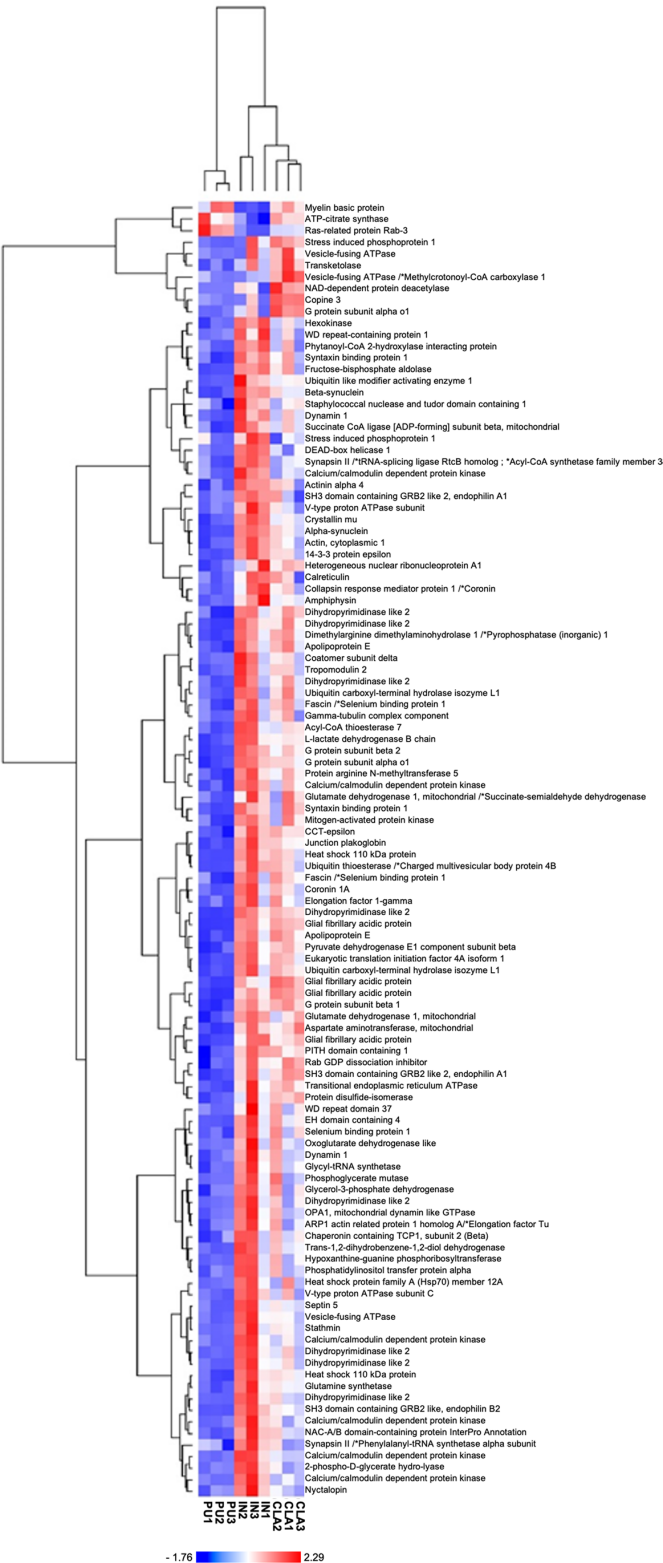
Clustered heat map of differentially expressed protein spots obtained by comparing different brain areas. The heat map was generated using the NG-CHM GUI 2.20.2 software. The Z-norm transform was used to normalize the row values (normalized spot optical densities, 3 samples from each brain area), and the resulting transform data matrix was used to build the heat map. A hierarchical ordering method was applied to clustered rows and columns. Euclidean distance metric was applied to hierarchically clustered rows and columns. Data matrix distribution values range from − 1.76 to + 2.29. The red color indicates increased expression whereas the blue color indicates decreased expression of proteins. Brighter colors indicate deeper changes. Protein names correspond to protein identifications shown in Tables 1, 2, and 3. *Spots with more than one identification

An exclusive expression difference was observed for sirtuin 2 (SIRT2), protein disulfide-isomerase (PDIA3), transketolase (TKT), and aspartate aminotransferase, mitochondrial (GOT2) in CLA vs PU but not in IN vs PU (Fig. 5B). Normalized mean values of optical density of identified differentially expressed spots were analyzed using next-generation clustered heat map to generate a clustered heat map (Fig. 6) where we can appreciate the highest consistence of protein expression increases as red color in IN followed by CLA and then PU.

**Fig. 7:**
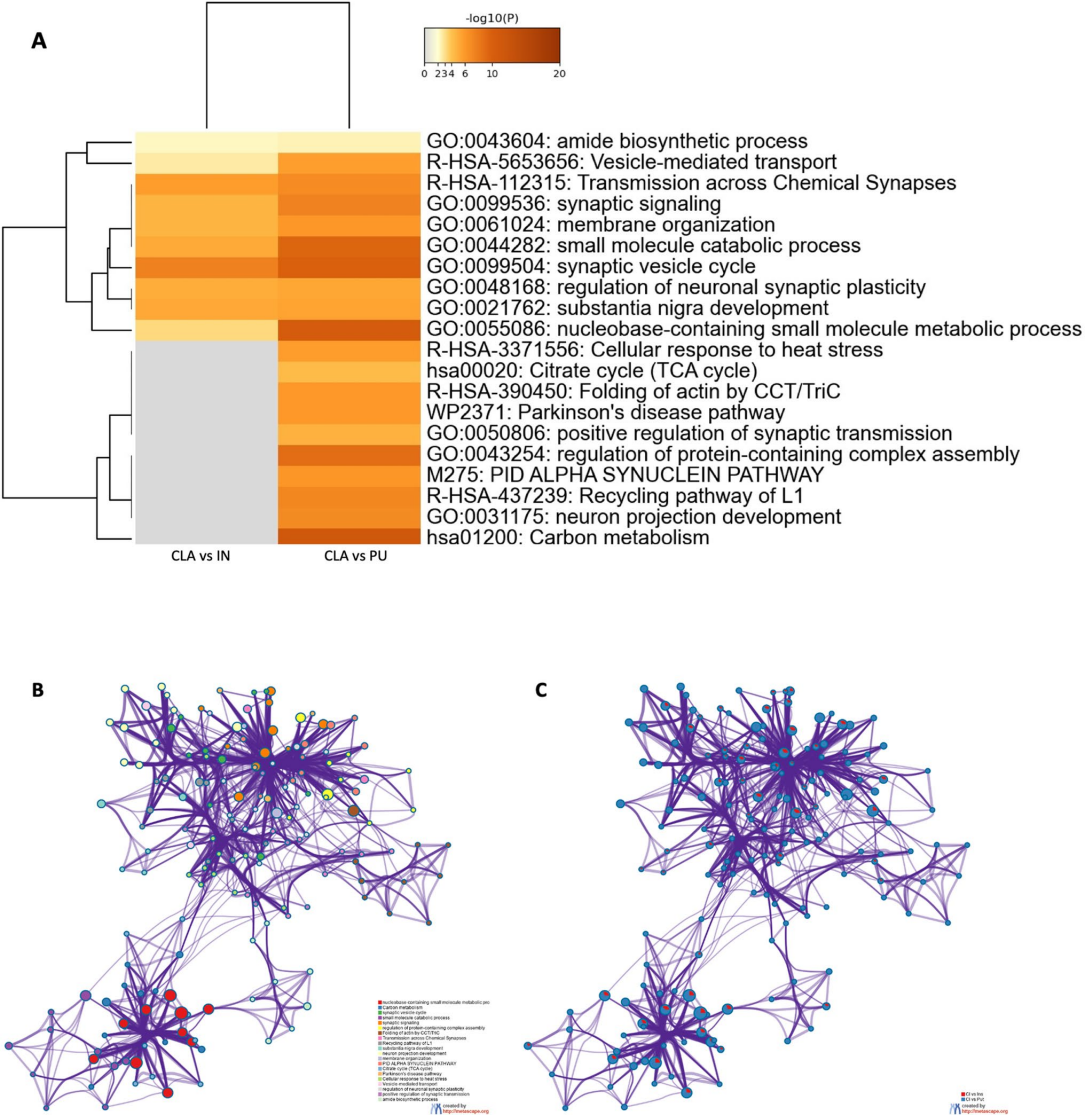
Visualization of meta-analysis results based on multiple gene lists. **A** Heat map showing the top enrichment clusters, one row per cluster. Statistical significance is represented using a discrete color scale while the gray color indicates lack of significance. Some categories are enriched only in one comparison (CLA vs PU), and therefore, processes associated with this gene list are likely, too. **B**, **C** Enriched network visualization of two gene list results. **B** Each term is represented by a circle node where its size is proportional to the number of input genes, which fall into that term, and its color represents its cluster identity (nodes of the same color belong to the same cluster). Terms with a similarity score > 0.3 are linked by an edge (the thickness of the edge represents the similarity score). **C** The same enrichment network with nodes displayed as pies. Each pie sector is proportional to the number of hits originated from a gene list. Color code for pie sector represents the identities of a gene lists: red (CLA vs IN) and blue (CLA vs PU)

The biological meaning of the differentially quantified proteins in two different comparisons (CLA vs PU and CLA vs IN) was investigated by a general overview using Metascape [36] which depicts top enriched clusters and their enrichment patterns across multiple gene lists as a clustered heat map (Fig. 7A). The heat map is complemented by an enrichment network (Fig. 7B) where each network node represents a category of biological processes and/or pathways. In Fig. 7C, each network node is represented by a pie chart, where the sector size is proportional to the number of genes originated from each gene list. Some categories such as PD pathway (WP2371) and α-synuclein pathway (M275) were enriched exclusively in CLA vs PU comparison and likely represent processes associated with proteins differentially expressed between these areas. The network also shows that processes such as synaptic vesicle cycle, regulation of neuronal synaptic plasticity, and substantia nigra development were shared between both lists.

**Fig. 8:**
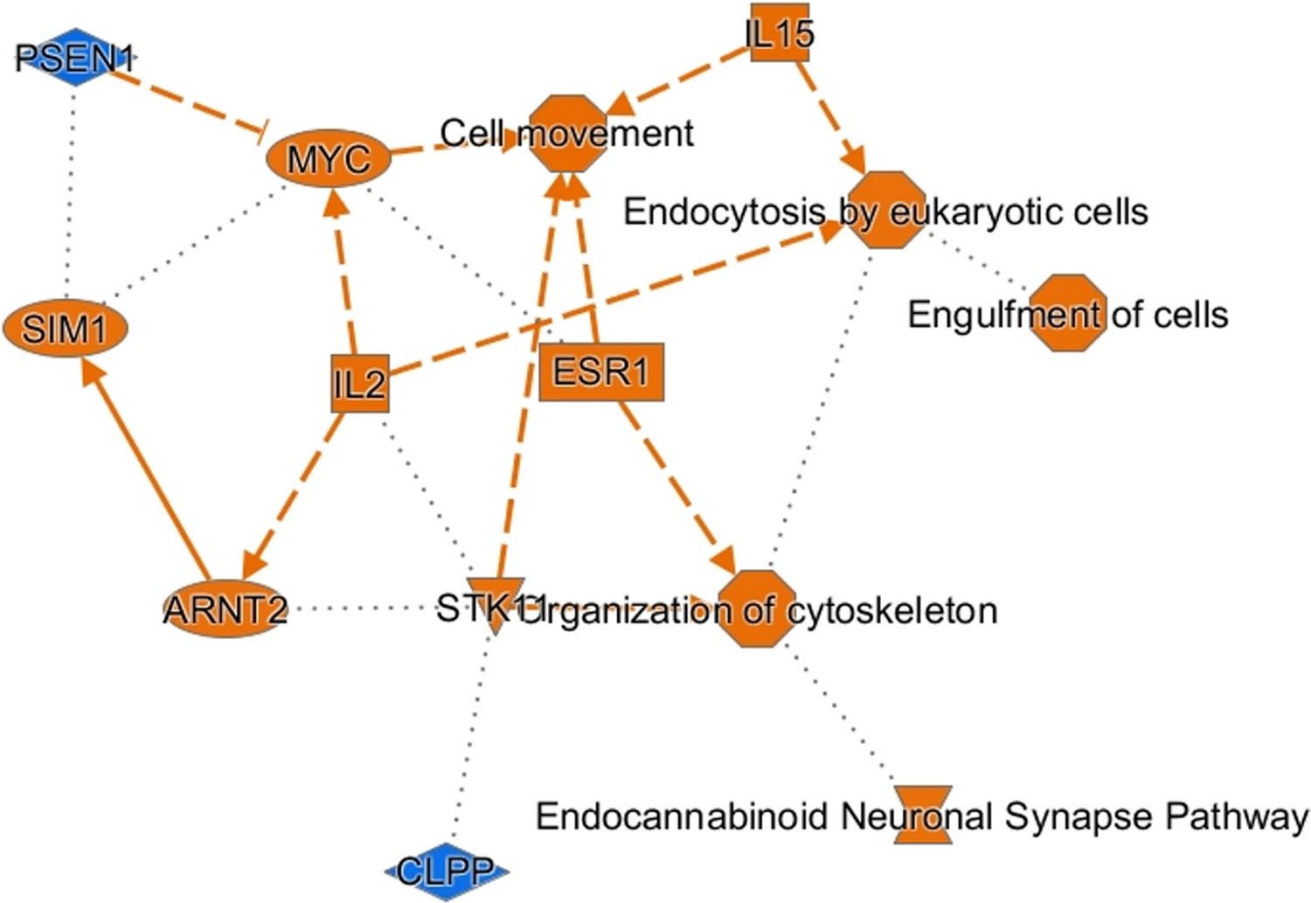
Synopsis originated by the IPA core analysis of differentially expressed proteins derived by comparing CLA vs PU. The graphical summary includes canonical pathways (endocannabinoid neuronal synapse pathway), upstream regulators (CLPP, ARNT2, STK11, SIM1, IL2, ESR1, MYC, IL15, and PSEN1), and biological functions (organization of cytoskeleton, engulfment of cells, endocytosis by eukaryotic cells, and cell movement). The orange color suggests activation whereas the blue color suggests inhibition. CLPP, ATP-dependent Clp protease proteolytic subunit, mitochondrial; ARNT2, aryl hydrocarbon receptor nuclear translocator 2; STK11, serine/threonine protein kinase 11; SIM1, single-minded homolog 1; IL2, interleukin 2; ESR1, estrogen receptor; MYC, Myc proto-oncogene protein; IL15, interleukin 15; PSEN1, presenilin-1

Proteins found differentially expressed in each different comparison were analyzed by IPA to discover the most enriched canonical pathways, possible upstream regulators, and downstream effects. Similarities between IN and CLA were evidenced by IPA results derived by comparing IN and CLA data with those of PU. In fact, the same most significant canonical pathways such as Parkinson’s signaling, synaptogenesis signaling, and insulin secretion signaling were generated. Figure 8 shows a coherent and comprehensible synopsis of IPA core analysis of CLA vs PU protein data set, so to obtain a quick overview of major biological themes and their relationships, the graphical summary includes a subset of the most significant canonical pathways, upstream regulators, diseases, and biological functions predicted by the analysis. Among the most significant regulators, inhibition of PSEN1 upstream regulator deserves to be underlined while activation of endocannabinoid neuronal synapse pathway is also worthy of attention.

### Western Blot Analysis

**Fig. 9:**
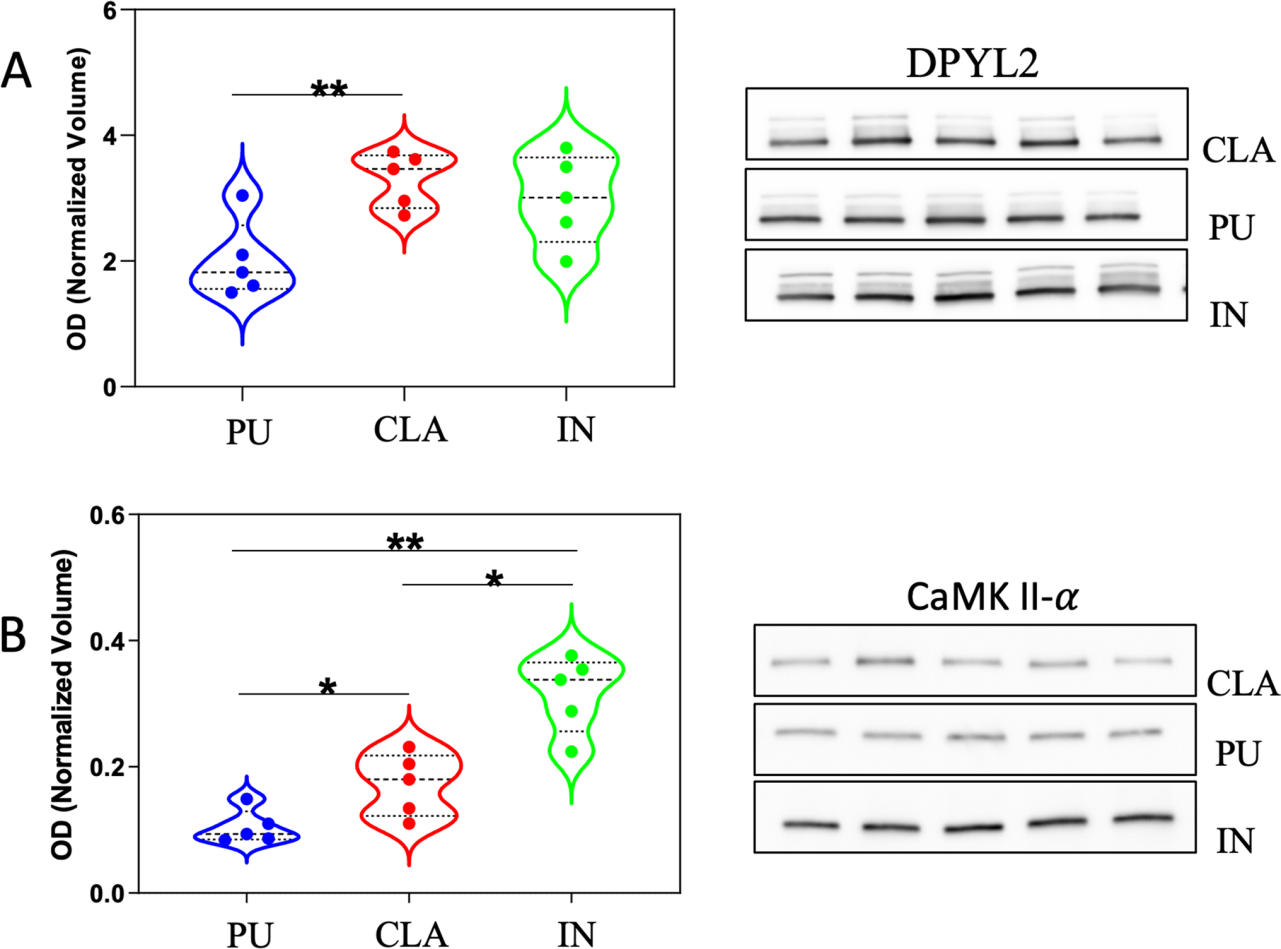
Validation of two differentially expressed proteins, DPYL2 and CaMKII-α, by western blot (WB) analysis. Violin plots of the normalized OD obtained in different brain areas are reported for DPYL2 (panel **A**, left) and CaMKII-α (panel **B**, left). Dashed lines represent the median whereas dot lines represent the first and the third quartiles. On the right (panels **A** and **B**), the immunoreactive bands of DPYL2 (main band of 65 kDa) and CaMKII-α (48 kDa) observed in five independent samples obtained from 5 different animals are shown. Statistical analysis was performed using a parametric paired *t* test. *p < 0.05, **p < 0.01

The difference of protein expression observed by 2-DE among three different brain areas was validated in other 5 additional samples by western blot analysis. The expression level of two more representative proteins, CAMK2 and DPYL2/CRMP-2, was detected using specific antibodies. A single immunoreactive band at 48 kDa was detected for CAMK2 whereas a main band at molecular weight of 65 kDa and two minor bands at 70 kDa and 75 kDa were detected for DPYL2 in agreement with different isoforms of this protein in *Sus scrofa*. Probably, the isoform with the highest apparent molecular weight represents the neuronal isoform with a ubiquitous localization in dendrites, axons, and cellular bodies [40]. Immunoreactive bands were analyzed, and normalized values of OD were represented in violin plots (Fig. 9A, B). The violin plot of DPYL2 shows the sum of all three immunoreactive band ODs. The expression differences observed by western blot analysis confirmed the results obtained by 2-DE according to which CLA expressed half levels of both proteins compared to IN (the highest level) and PU.

### Immunofluorescence

**Fig. 10:**
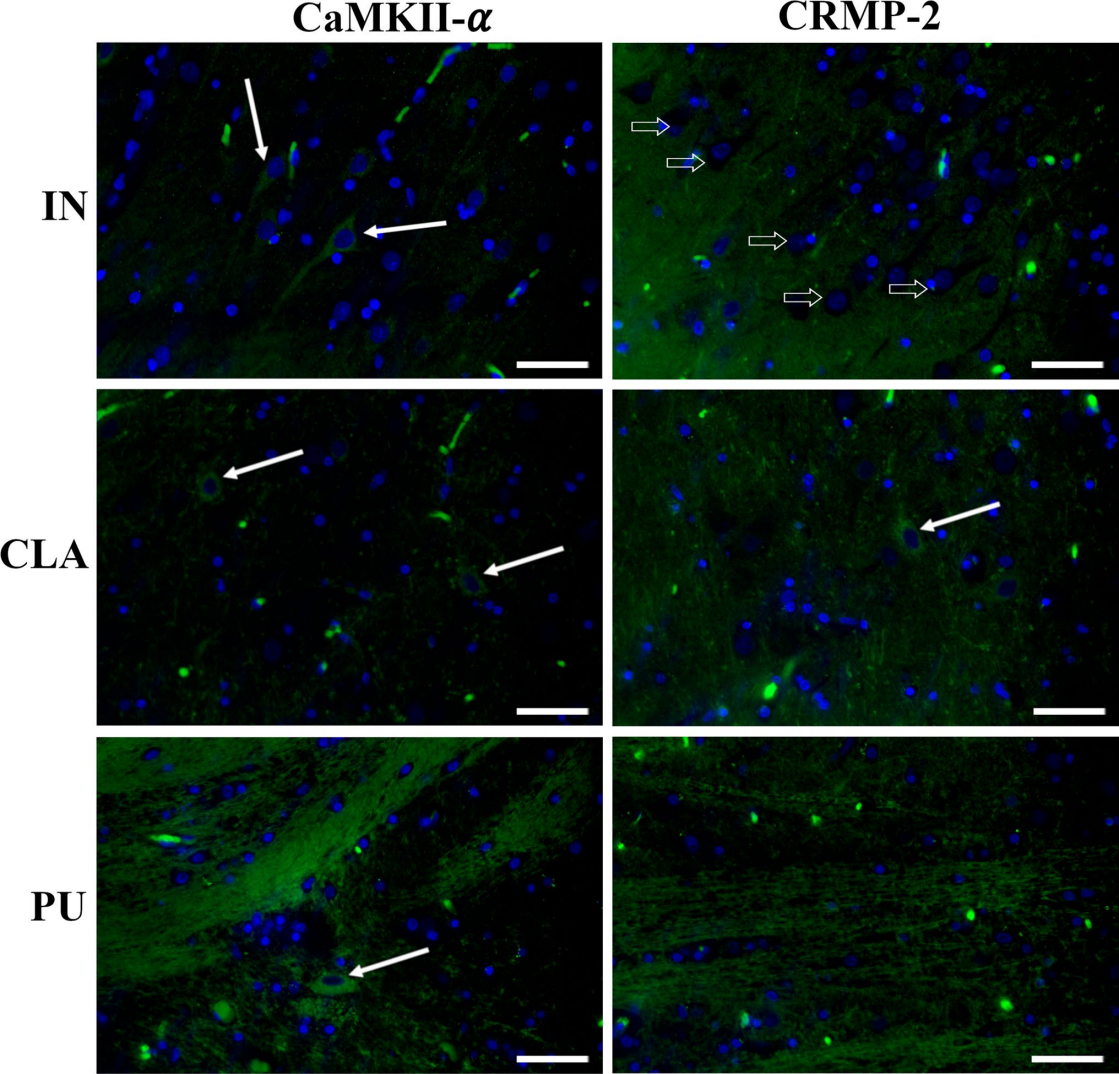
Immunofluorescent staining of pig insula (IN), claustrum (CLA), and putamen (PU). In IN, CaMKII-α was mainly localized in neuron somas (white arrows) while CRMP-2 labeling was mostly associated with neuropil surrounding negative cell bodies (white arrows). A similar immunostaining pattern was observed in CLA, which also showed rare CRMP-2-positive somas (white arrow). In PU, positivity to both CaMKII-α and DPYL2/CRMP-2 was localized in fibers while rare immunolabeled cell bodies (white arrow) were only seen for CaMKII-α. Scale bars = 50 μm

Immunofluorescent staining in pig CLA, PU, and IN revealed the presence of both CaMKII-α and DPYL2/CRMP-2 (Fig. 10). In the IN, CaMKII-α was mainly localized in neuron somas while DPYL2 labeling was mostly associated with neuropil surrounding negative cell bodies. A similar immunostaining pattern was observed in CLA where few DPYL2-positive somas were also found.

On the contrary, in PU, positivity to both CaMKII-α and DPYL2/CRMP-2 was localized in fibers while rare immunolabeled cell bodies were only seen for CaMKII-α.

## Discussion

Over the past decades, because of its considerable resemblance to human anatomy and physiology, pig brain has been widely employed as a valuable model in biomedical studies [28]. Recently, the generation of genetically modified pig models of neurodegenerative disorders has been discussed [41]. In the present study, we carried out a comparative proteomic analysis of pig CLA, IN, and PU to reveal specific molecular hallmarks of CLA to clarify its role, origin, and possible implication in human neurological disorders.

Although the three areas showed superimposable 2-DE protein maps, a considerable divergence in protein expression level was observed between CLA and IN when compared to PU; in this context, the circos plot showed that CLA assumed an intermediate position with respect to both IN and PU.

The ontology of the claustro-insular complex is still subject to debate [15, 42], and homologies reaching birds and even reptiles have been put forward [43, 44]. According to Bruguier et al. [18], the claustral neuronal population is born first starting from the lateral pallium, then insular cells migrate radially through the CLA, occupying progressively more superficial positions; thus, IN development is linked to CLA. In line with this hypothesis, the minor differences of protein expression observed between CLA and IN seem to support their common origin in agreement with described morphogenetic and neurochemical similarities of these structures [15, 16].

In general, we found PU to show a lower level of differentially expressed proteins except for Rab3A that showed a very significant high level of expression. Traditionally, PU is involved in different functions such as learning and motor control, reward, cognitive functioning, and addiction [45–49]; moreover, it appears to be correlated with a broad spectrum of movement disorders including PD, and Huntington’s disease (HD) as well as psychiatric diseases such as schizophrenia or obsessive–compulsive disorder (OCD) [50–55]. Rab proteins are small GTPases involved in all stages of vesicular transport and membrane fusion in mammalian cells, and Rab3 isoforms (Rab3A, Rab3B, Rab3C, and Rab3D) are expressed almost exclusively in neurons and secretory cells and are mainly located at synaptic membranes regulating Ca^2+^-dependent neurotransmitter release [56]. Rab3A has been indicated as one of physiological substrates of leucine-rich repeat kinase 2 (LRRK2) whose increased activity is related to PD pathogenesis [57]. In light of the functions ascribed to Rab3A, the high expression of Rab3A in pig PU adds molecular evidence in support of the potential role of this brain area in PD pathogenesis as prompted by different approaches such as shape analysis [58], and it is worth noting that Rab3A expression was also elevated in CLA with a value significantly higher than in IN, suggesting a possible common role of PU and CLA in neurological disorders.

Exclusive expression differences were observed for SIRT2, PDIA3, TKT, and GOT2 in CLA vs PU. SIRT2 belongs to mammalian sirtuin family that consists of seven members (SIRT1–SIRT7) with diverse functions depending upon substrates, distinct subcellular localization, and expression patterns [59]. SIRT2 is localized in the cytoplasm of both neurons and oligodendrocytes [60], and the well-known substrate of SIRT2 is α-tubulin, an important component of microtubule cytoskeleton whose acetylation by SIRT2 is linked to brain aging and neurological disorders [61] such as AD [62, 63] and PD [64, 65]. Similarly, PDIA3 is indicated as potentially involved in neurodegeneration processes. The protein disulfide-isomerases (PDIs) are generally localized to the endoplasmic reticulum (ER) where they mediate thiol–disulfide interchanges, which is a critical process during post-translational protein folding [66], and PDIA3 is markedly upregulated in most common neurodegenerative diseases, highlighting ER as an emerging driver of neurodegeneration [67, 68]. Also, TKT, a multifunctional protein in the non-oxidative branch of the pentose phosphate pathway, seems to be related to some neurological disorders such as AD, PD, and Wernicke-Korsakoff syndrome, and reduced levels of TKT have been found in the substantia nigra of PD patients [69].

CLA neurodegeneration and dysfunction are described in patients affected by AD or PD [70, 71]. Aggregation of misfolded proteins is a determinant in many neurodegenerative diseases such as frontotemporal dementia (FTD), amyotrophic lateral sclerosis (ALS), PD, AD, and HD [72], and amyloid β (Aβ) deposits and neurofibrillary tangles have been described in the CLA of AD patients [73, 74]. In addition, α-synuclein and Aβ lesions have been also found in the CLA of cases affected by PD or dementia with Lewy bodies (DLB) [75] while claustral degeneration has been reported in familial Alzheimer’s disease [71]. In the present study, Metascape analysis of CLA vs PU suggested the activation of α-synuclein and PD pathways, corroborating the implication of these structures in the abovementioned diseases. Two other interesting pathways evidenced by Metascape analysis were the neuron projection development pathway and L1 recycling pathway; the first is involved in axonal growth while the second plays a role in clathrin-coated vesicle trafficking. The endocytic recycling pathway seems to be implicated in the aggregation, toxicity, and secretion of α-synuclein, whose misfolding is common in several neurodegenerative diseases [76]. Among CLA differentially expressed proteins involved in these pathways (Supplementary Table 1), we also found DPYSL2 and CAMK2, and the expression of both proteins was validated by western blot analysis while immunofluorescence revealed their distribution and cellular localization in the three cerebral structures. CAMK2 plays a key role in the redistribution of α-synuclein during neurotransmitter release [77] and can interact and potentially alter α-synuclein conformation [78] whereas DPYSL2 has a function in neuronal development and polarity, in cell migration and endocytosis, and is implicated in neurological disorders like CAMK2 [79].

Another interesting protein overexpressed in CLA compared to PU is aspartate aminotransferase 2 (GOT2), a pyridoxal 5′-phosphate (PLP)-dependent enzyme that exists as cytosolic (GOT1) and intramitochondrial (GOT2). GOT2 deficiency is a mitochondriopathy that is reported to be implicated in treatable metabolic epilepsies [80]. In both humans and rodents, CLA seems to be a potential locus for generating epileptiform activity during kindling [81], and GOT2 overexpression found in pig CLA strengthens the assumption that depicts this structure as involved in seizure generation.

Our findings revealed that copine 3 (CPNE3) and MBP were overexpressed in CLA compared to IN. Copines are calcium-dependent phospholipid-binding proteins involved in membrane-trafficking phenomena and protein–protein interactions [82]. A recent study reported that CPNE3 interacts with anxiety to affect working memory (WM) [83]. WM is a cognitive ability that allows one to hold and manipulate information and is foundational to the organization of goal-directed behavior [84]. Goll et al. [2] have proposed that CLA has the capacity to focus attention, an essential function for goal-direct behavior; besides, White et al. [3] have provided data describing CLA as an anatomical and functional substrate that may underlie functions, such as executive attention or WM. Thus, such reported findings support our data regarding CPNE3 and, taken together, may indicate the CLA as a possible site of mutual influences between anxiety and working memory.

In psychiatric disorders such as schizophrenia and attention deficit hyperactivity disorder, WM impairment is reported [83], and in this context, our data showing an overexpression of MBP in CLA are quite interesting. MBP is important in maintaining the structure of the myelin sheath [85]. Myelin dysfunction produces abnormal connectivity of neural networks and is considered one of the main factors implicated in schizophrenia pathogenesis [86, 87]. Different studies have shown structural differences in the CLA of schizophrenic patients experiencing delusions [88, 89].

Last but not the least, protein data set analysis of CLA vs PU comparison by IPA produced an intriguing result highlighting the activation of endocannabinoid neuronal synapse pathway. The endocannabinoid system (ECS) is a widespread neuromodulator network involved in central nervous system development and plays a major role in tuning many cognitive and physiological processes [90]. The activation of endocannabinoid neuronal synapse pathway revealed by IPA analysis supports previous immunohistochemical studies demonstrating the presence of cannabinoid receptor 1 (CB1) and two endogenous cannabinoid-degrading enzymes (MGL and FAAH) in pig and dog CLA [91, 92]. Taken together, the whole findings provide a neuroanatomical support for a possible neuromodulator role of endocannabinoids within CLA circuitry and may reinforce the postulate depicting CLA and ECS as involved in neurodegenerative diseases [70, 93].

## Conclusion

In conclusion, the comparative study of the proteomic profile of pig CLA highlighted the overexpression of specific proteins deeply implicated in both neurodegenerative (e.g., PD, AD, and HD) and psychiatric disorders in humans, pointing out the translational significance of the investigation. In this context, the present findings may contribute to better understand the molecular involvement of CLA in the pathogenetic mechanisms of these diseases. Furthermore, the minor differences of protein expression observed between CLA and IN strengthen the hypothesis of their common origin.

### Supplementary Information

ESM 1 Supplementary Table

## Data Availability

All data generated or analyzed during this study are included in this published article.
