# Correction: Proteomic Profiling Reveals Specific Molecular Hallmarks of the Pig Claustrum

**DOI:** 10.1007/s12035-024-04165-w

**Published:** 2024-04-06

**Authors:** Andrea Pirone, Federica Ciregia, Giulia Lazzarini, Vincenzo Miragliotta, Maurizio Ronci, Mariachiara Zuccarini, Lorenzo Zallocco, Daniela Beghelli, Maria Rosa Mazzoni, Antonio Lucacchini, Laura Giusti

**Affiliations:** 1 Department of Veterinary Sciences, University of Pisa, Pisa, Italy; 2 Department of Clinical and Experimental Medicine, University of Pisa, Pisa, Italy; 3 Department of Medical, Oral and Biotechnological Sciences, University G. D’Annunzio of Chieti-Pescara, Chieti, Italy; 4 Interuniversitary Consortium for Engineering and Medicine, COIIM, Campobasso, Italy; 5 Department of Translational Research and New Technologies in Medicine and Surgery, University of Pisa, Pisa, Italy; 6 School of Biosciences and Veterinary Medicine, University of Camerino, Camerino, Italy; 7 Department of Pharmacy, University of Pisa, Pisa, Italy; 8 School of Pharmacy, University of Camerino, Camerino, Italy

**Correction to: Molecular Neurobiology (2023) 60:4336-4358** 10.1007/s12035-023-03347-2

The original version of this article unfortunately contained an error. Specifically, in the list of authors, the last author’s name and surname have been switched. It should be from “Giusti Laura” to “Laura Giusti.”

The original article has been corrected.

